# Utility of Ultrasound in Evaluating an Ambiguously Working Thoracic Epidural: A Case Series

**DOI:** 10.7759/cureus.71916

**Published:** 2024-10-20

**Authors:** Alexander Ip, Anthony Barisano, Vendhan Ramanujam

**Affiliations:** 1 Anesthesiology, Rhode Island Hospital, Brown University, Providence, USA

**Keywords:** acute pain services, anesthesiology, regional anesthesiology, thoracic epidural analgesia, ultrasonography (us), ultrasound in anesthesiology

## Abstract

A thoracic epidural is commonly used for analgesia following trauma or surgery. Traditionally, thoracic epidurals are performed using the loss of resistance technique. However, false-positive loss of resistance and catheter placement outside the epidural space is possible. In such scenarios and when clinical equivalents, such as hemodynamic changes and sensory loss to epidural blockade, fail as well, assessment of the catheter position can be accomplished using ultrasound. Ultrasound scanning can identify the catheter and spread of the injectate past the laminas to confirm its epidural positioning. In addition, multiple scanning approaches can be used to view the interlaminar spaces at the thoracic level, which are usually challenging to visualize compared to the lumbar level to verify the epidural catheter. It can be done at the bedside, in real-time, and with the feasibility of obtaining immediate results. This way, a nonfunctioning catheter can be replaced promptly to achieve adequate analgesia. We present a case series of patients in whom an ultrasound was utilized to evaluate and replace ambiguously working thoracic epidural catheters that were primarily placed by the loss of resistance technique.

## Introduction

A thoracic epidural is a commonly used pain management technique where local anesthetics are administered continuously through a catheter placed in the epidural space to provide analgesia for both trauma and surgery involving the thorax and abdomen. They are popular since they provide superior analgesia compared to opioids, decrease respiratory complications, and promote early mobility [1,2]. Like any other intervention, there are associated risks that include inadequate analgesia due to catheter failure, which has a prevalence between 12% and 50% [3-5]. Among the several causes of catheter failure, failure to place it in the epidural space during the primary attempt is an important contributing factor, and it has been reported to be up to 15% [6]. This results in undesirable complications such as patient discomfort and inadequate analgesia. Recently, ultrasound has gained attention as a point-of-care tool for guiding epidural placement [7,8]. Since visualization of the catheter tip and epidural spread of the injectate under ultrasound confirms the epidural catheter placement, the same can be used to evaluate a previously placed epidural to ascertain its position. The current literature is sparse on reporting this utility. We present a case series of patients in whom we used ultrasound to evaluate thoracic epidural catheters primarily placed by the loss of resistance (LOR) technique when their functioning was found to be uncertain. This report can be useful for assessing ambiguously working thoracic epidurals using ultrasound at the bedside and managing them accordingly to achieve adequate pain control.

## Case presentation

As the case series is devoid of patient-identifiable information, it was exempt from institutional review board review requirements as per Lifespan policy. Nevertheless, patient informed consent was obtained for submission. This manuscript adheres to the case reports (CARE) guidelines. This report includes four patients in whom thoracic epidural catheters were placed either for post-surgical pain control or post-trauma rib fracture pain control. None of the patients had any previous history of chronic pain or opioid use. All the epidurals were performed by the LOR technique under American Society of Anesthesiologists (ASA) monitoring and sterile precautions [9]. Resident trainees performed them under the supervision of an attending anesthesiologist. A 17-gauge x 8.57-cm Tuohy needle and 19-gauge x 90-cm single, open-end hole catheter (Arrow^®^, FlexTip Plus^®^, Morrisville, USA) were utilized for this purpose. After an LOR was attained at a certain needle depth, the catheters were advanced and secured at a length of 5 cm in addition to the LOR depth. After testing the catheters for negative aspiration of either cerebrospinal fluid or blood and negative effect after administration of a test dose of 3 ml of lidocaine with epinephrine (preservative free) 1.5% - 1:200,000, the catheters were secured and an infusion was started. Except for failed analgesia following the primary placement, there were no other associated complications. When the epidural analgesia was inadequate following the primary placement, maneuvers such as evaluation of the catheter securement site to rule out migration and increasing the dose of local anesthetic through bolus and continuous infusion in the catheter to achieve adequate levels of blockade were attempted. When these attempts failed, the epidural catheters were evaluated using a high frequency (6-13 Megahertz) curvilinear probe of an ultrasound (Fujifilm SonoSite, Inc., Bothell, Washington, USA) either in parasagittal oblique or transverse view by another anesthesiologist. For the parasagittal oblique view, from the site of catheter insertion, the transducer was initially placed approximately 5 cm lateral in a craniocaudal orientation, either on the left or right side to identify ribs as a hyperechoic bordered structure with shadowing below and pleura in the intercostal space. The transducer was slid medially to identify the transverse process as a squarer and deep-lying hyperechoic bordered structure, then the articular process as a continuous hump-like hyperechoic structure, and finally, the lamina as a non-continuous sawtooth-like hyperechoic structure. A slight medial tilt was then performed to view the interlaminar acoustic window to visualize either or both the posterior complex (ligamentum flavum, posterior dura) and anterior complex (anterior dura, posterior longitudinal ligament, vertebral body) of the thoracic spine. For the transverse view, from the site of catheter insertion, the transducer was initially placed in a transverse orientation, either above or below to identify the spinous process as a superficially lying hyperechoic structure with shadowing beneath and a sloping hyperechoic lamina bilaterally. Then, the transducer was slid either cephalad or caudad to view the interlaminar space for the visualization of either or both posterior and anterior complexes along with laterally lying hyperechoic articular and transverse processes. Video 1 demonstrates the ultrasound scanning technique for both views.

**Video 1:** Ultrasound scanning techniques for the spine during thoracic epidural catheter evaluation

During scanning in both these views, the catheter was identified as a hyperechoic linear structure that was able to be tracked from the level of the skin. After selecting the best planar and interlaminar window view, where either the entire or a part of the catheter was able to be viewed, an injection of 5 to 10 ml of normal saline was injected via the catheter and its flow beyond the tip was directly visualized. Visualization of the catheter tip and flow of the injected saline outside the interspace of laminas was identified as failed catheter placement outside the epidural space.

Case 1

**Figure 1:**
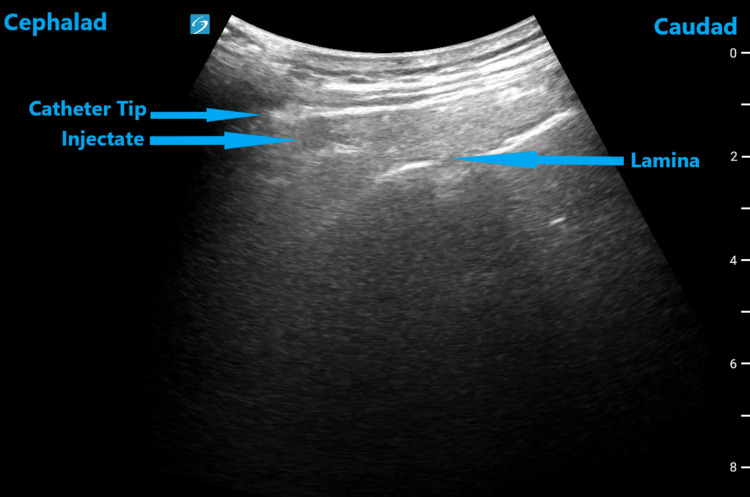
Ultrasound image of the thoracic spine in the parasagittal oblique view demonstrating the catheter outside the epidural space

A 65-year-old female with a body mass index (BMI) of 48.8 kg/m^2^ and an ASA III classification received a thoracic six-seven level epidural catheter for post-surgical pain control on day one after the surgery. She underwent an elective anterior chest wall resection and reconstruction for sarcoma. After surgery, she remained intubated and was admitted to the intensive care unit. On the second day after surgery, she was extubated and later re-intubated for increased work of breathing due to inadequate pain control. Her past medical history was significant for morbid obesity, obstructive sleep apnea, hypertension, diabetes, adrenal insufficiency, thyroid, and breast cancer. She was allergic to penicillin. She was on a continuous epidural infusion of 0.06% bupivacaine, intravenous acetaminophen, and fentanyl for pain control. On examination, her vital signs and laboratory values were within normal limits. She was on ventilatory support and remained sedated with continuous intravenous propofol. The re-intubation and failed catheter troubleshooting maneuvers prompted its further evaluation with an ultrasound. With the patient in lateral decubitus position, using an ultrasound parasagittal oblique view, the catheter was evaluated and found to be outside the epidural space (Figure 1). Hence, it was discontinued, and a new catheter was placed at the same level using the same technique. This time, a reliable epidural blockade and good pain control were achieved with a continuous epidural infusion of 0.1% bupivacaine. It was continued for the next seven days. The patient was extubated and transitioned to oral acetaminophen, oxycodone, and transdermal fentanyl during this period. After a few weeks, she was discharged from the intensive care unit.

Case 2

**Figure 2:**
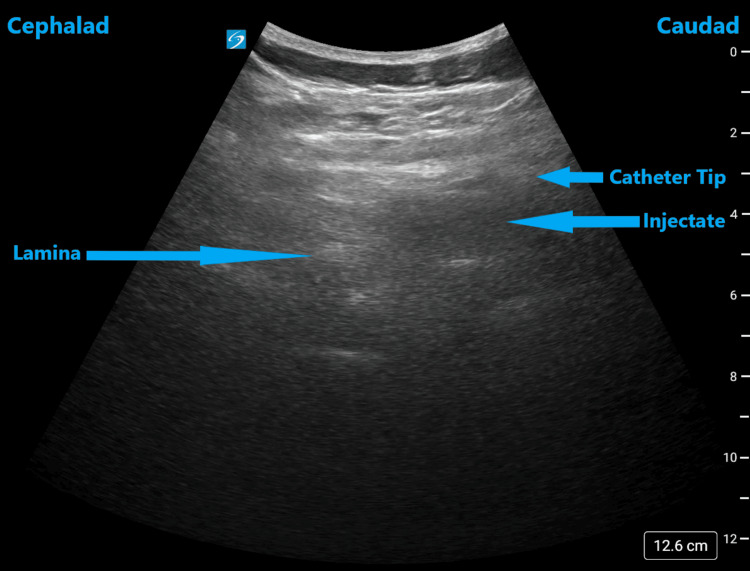
Ultrasound image of the thoracic spine in parasagittal oblique view demonstrating the catheter outside the epidural space

An 80-year-old male with a BMI of 28.18 kg/m^2^ and an ASA III classification had an epidural placed at the thoracic five-six level for post-rib fracture pain control. He was admitted with multiple right-sided rib fractures from two to seven following a fall. His past medical history included mild cognitive decline, hypertension, and hyperlipidemia. He denied any known drug allergies. He was on a patient-controlled epidural analgesia with 0.1% bupivacaine, oral acetaminophen, and intravenous hydromorphone for pain control. On examination, his oxygen saturation was 92% while being on 2 liters of supplemental oxygen. He had reduced breath sounds on the right side. The rest of the examination and laboratory values were unremarkable. The day after placement, he continued to have poor pain control with increasing oxygen requirement. Suspecting failed epidural analgesia, all the correction maneuvers were performed but failed. An ultrasound evaluation of the catheter was then performed in parasagittal oblique view with the patient in a sitting position. The catheter tip and injectate spread were witnessed outside the epidural space (Figure 2). Hence, it was discontinued and replaced at the same position. A consistent epidural blockade and pain control were then achieved. It was continued for the next three days with the previous epidural analgesia regimen. He was able to complete chest physiotherapy and was discharged the day after catheter discontinuation with oral acetaminophen and tramadol for pain control.

Case 3

**Figure 3:**
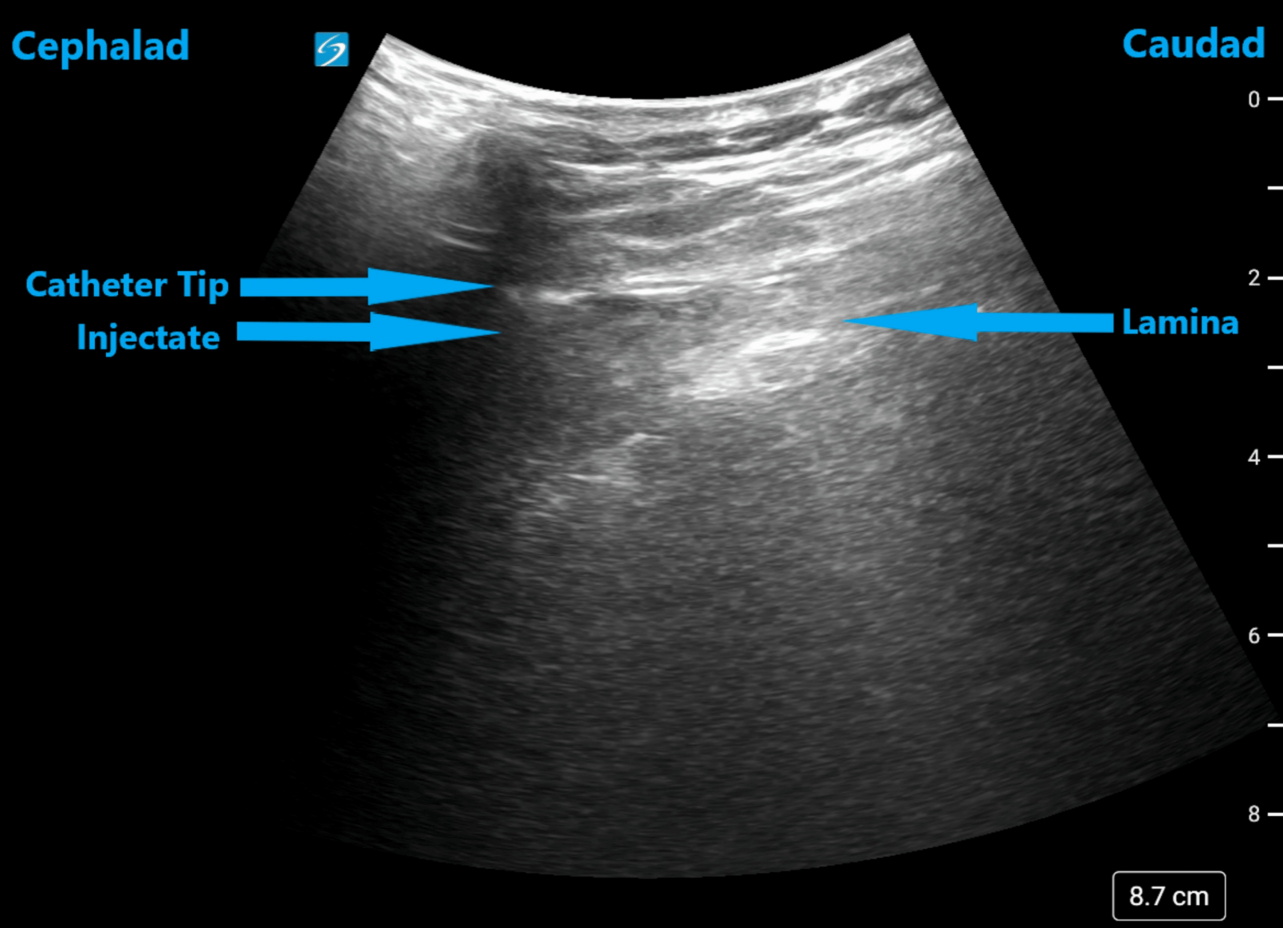
Ultrasound image of the thoracic spine in the parasagittal oblique view demonstrating the catheter outside the epidural space

A 52-year-old female with a BMI of 21.7 kg/m^2^ and ASA II classification had a thoracic epidural placed at the thoracic 10-11 level immediately after her explorative laparotomy with a total abdominal hysterectomy and bilateral salpingo-oophorectomy surgery for pain control. She had a past medical history of uterine cancer, idiopathic thrombocytopenic purpura, and anxiety. She was allergic to penicillin. She was on a patient-controlled epidural analgesia with 0.06% bupivacaine, oral acetaminophen, and intravenous hydromorphone for pain control. On examination, her vital signs and laboratory values were within normal limits. On the first and second days after surgery, she had inadequate pain control, and during the examination, reported an inconsistent loss of sensations only over the left thoracoabdominal region, which did not improve despite all the readjustment maneuvers. Hence, an ultrasound evaluation of the catheter was done with the patient in a sitting position and along the parasagittal oblique view, which revealed that the catheter tip was not in the epidural space (Figure 3). It was subsequently replaced at the thoracic eight-nine level with satisfactory blockade and pain control. It was used for the next couple of days with the previous regimen until the patient was able to transition to oral acetaminophen and oxycodone. She was later discharged home the day after the catheter was discontinued.

Case 4

**Figure 4:**
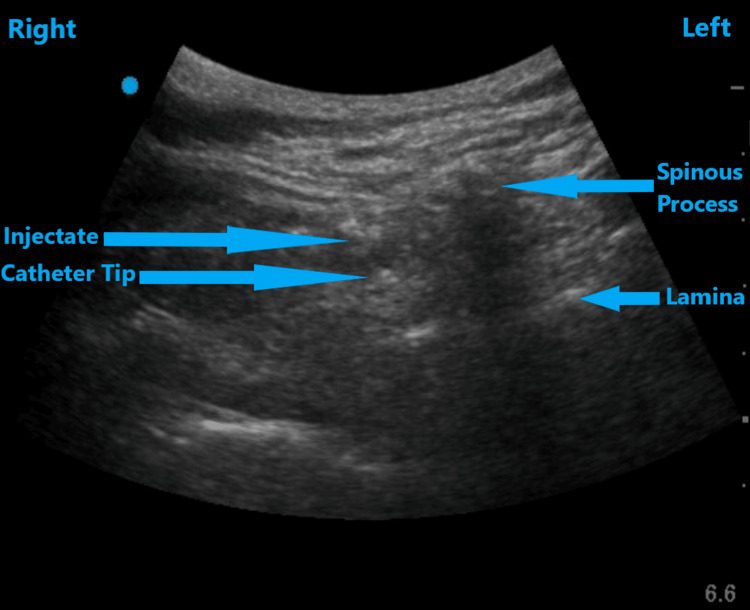
Ultrasound image of the thoracic spine in transverse view, demonstrating the catheter outside the epidural space

An 85-year-old female with a BMI of 29.3 kg/m^2^ and an ASA II classification received a thoracic 8-9 level epidural catheter before her explorative laparotomy with resection of a large retroperitoneal liposarcoma for post-surgical pain control. Her past medical history was significant for hypertension and hyperlipidemia. She had no known drug allergies. She was on a continuous epidural infusion of 0.06% bupivacaine and as needed intravenous hydromorphone for pain control in the post-anesthesia care unit. On examination, her heart rate was elevated. The rest of the examination and laboratory values were within normal limits. Her pain was poorly controlled and no loss of sensations was detected in the thoracoabdominal region during evaluation. It did not improve even after the rectifying maneuvers. This directed the ultrasound evaluation of the catheter with the patient in a sitting position. Since the catheter was not visualized under a parasagittal oblique view, a transverse view was utilized where it was found to be outside the epidural space (Figure 4). It was replaced at the same level, which immediately started working to achieve good pain control. It was used for the next three days before discontinuation. She was able to take oral acetaminophen and tramadol for pain control and was discharged home the day after that.

## Discussion

Epidural provides better and safer analgesia compared to systemic medications in post-surgical as well as post-rib fracture pain management [10,11]. The placement of an epidural catheter has traditionally been accomplished by the LOR technique [9]. After sequentially passing the epidural needle through the skin, supraspinous ligament, interspinous ligament, and ligamentum flavum, needle entry in the epidural space leads to a LOR at the syringe that is attached to a needle filled with either saline or air. However, a false loss of resistance can also happen before the needle enters the epidural space when it penetrates cavity spaces in the paravertebral muscles or within the degenerated ligaments. This leads to the placement of the epidural catheter outside the epidural space. With the LOR technique lacking specificity and prone to variability between different performers, clinical parameters, such as a decrease in blood pressure and loss of sensations to cold and pain following an epidural blockade, are usually used to confirm the correct position and functioning of the epidural catheter. This can be challenging to verify when patients are under or recovering from anesthesia or trauma where they can be either uncooperative or unreliable. There can be catheter migration out of the epidural space after initial placement that can contribute to their failure as well. In such scenarios, objective tests such as epidurography, electrical stimulation, and waveform analysis can be useful, but they are limited due to unique equipment requirements [12].

Ultrasound is a bedside tool used for several diagnostic and therapeutic purposes [13]. Recently, it has become popular in assisting epidural catheter placements by improving procedural performance without affecting success rates as compared to landmark and fluoroscopy-based techniques [4,14-16]. This is due to the potential of ultrasound imaging to recognize finer and more accurate neuraxial structures, such as the spinous process, lamina, ligamentum flavum, and posterior dura, which guide the epidural needle insertion and catheter placement in the epidural space [17]. A systemic approach to scanning as described in this report is key to identifying relevant structures and, ultimately, the epidural catheter’s position. Due to caudad angulation of the spinous process and overlying lamina in the thoracic spine in comparison to the lumbar spine, ultrasound scanning and imaging can still be difficult as seen in these cases. Yet, the chances of obtaining an interlaminar window to view the epidural space may be better with the parasagittal oblique view as compared to the transverse view, which can be considered an alternate when the initial imaging through the parasagittal oblique view fails. After catheter placement, saline injection through the catheter helps confirm its epidural position in both parasagittal oblique and transverse views by demonstrating a downward shift of the posterior dura while failed placement will demonstrate bulging around the lamina without the downward shifting of the posterior dura. The same can be used in evaluating dysfunctional catheters whose epidural position is suspicious as seen in the patients reported here. A recent investigation in the obstetric population revealed the feasibility of ultrasound in evaluating the labor epidural catheters placed in the lumbar region [18]. Although ultrasound scanning of the thoracic spine can be challenging, as described earlier, using both the parasagittal oblique and the transverse scanning views can help detect the thoracic epidural catheters as demonstrated in this report. This can also be enhanced by using the color Doppler effect while injecting saline through the catheter to visualize the changes in color in the ultrasound image [18,19]. The other advantages of using ultrasound for evaluating epidural catheters include real-time examination to provide immediate results, easy availability of equipment, feasibility to be used anywhere, and ability to perform the investigation in any position [17]. The disadvantages can be difficulty in consistently visualizing the catheter tip especially if it had exited through the transvertebral foramen, distorted images from previously injected fluid or air, and variability in interpretation due to the experience of the provider performing the scan [17]. 

This report has limitations. This is a case series, and the findings are retrospective, non-comparable to other diagnostic interventions, and might be over-interpreted. Hence, ultrasound evaluation of thoracic epidural catheters to determine their functionality cannot be generalized and validated.

## Conclusions

To conclude, the ultrasound is a useful tool for evaluating thoracic epidural catheters at the bedside whose functioning is uncertain. It can be done using both parasagittal oblique and transverse scanning views with the patient in a sitting or lateral decubitus position. Since visualizing the catheter alone can be challenging, saline injection and evaluation of its spread with or without color Doppler can be effective in determining the catheter position. This can help in mending a failed catheter to achieve adequate pain control. Immediate evaluation of a challenging or uncertain placement can salvage unnecessary patient discomfort. Focused training and regular practice with this technique will be essential to its success. This is only a case series; hence, future studies are needed to evaluate and validate their use.
